# Spatial distribution of neighborhood-level housing prices and its association with all-cause mortality in Seoul, Korea (2013–2018): A spatial panel data analysis

**DOI:** 10.1016/j.ssmph.2021.100963

**Published:** 2021-11-11

**Authors:** Ikhan Kim

**Affiliations:** Department of Medical Humanities and Social Medicine, Kosin University College of Medicine, 262 Gamcheon-ro, Seo-gu, Busan, 49267, South Korea

**Keywords:** Housing, Health status disparities, Residence characteristics, Socioeconomic factors, Spatial analysis, Seou

## Abstract

Housing prices are known to be a relevant indicator of the socioeconomic position of the neighborhood. In a society where the market system mainly drives housing prices, residents' spatial patterning is formulated according to their socioeconomic position. Dividing the 2013–2018 entire study period into three periods, we explored the spatial distribution of housing prices and all-cause mortality and their association in Seoul, the country's capital city. The government authorities' data and 2015 census data were used for the study. We mapped the spatial distribution of housing prices and all-cause mortality and investigated the changes in distribution. We conducted a pooled ordinary least square (OLS) and spatial panel regression analysis to estimate housing prices elasticity of all-cause mortality. We also explored the possible mediating role of housing prices on the educational composition's effect on all-cause mortality. We found the common trends of increasing spatial patterning of housing prices and all-cause mortality. The magnitude of spatial patterning was far greater in housing prices than all-cause mortality. A pooled OLS regression analysis found that a 1% increase in housing price was associated with a 0.11% reduction in all-cause mortality after controlling the explanatory variables. Attenuation in the regression coefficient's magnitude was found after adding the neighborhood's educational composition to the model. As a result of spatial panel analysis, we found a direction and scale similar to the housing price elasticity of all-cause mortality in the final pooled OLS model. The results suggested that spatial health inequality in Korea's urban space mainly stems from socioeconomic inequality.

## Highlights

• Korea's housing system is highly market-driven, and the distribution of housing prices is spatially patterned.
• Doubling the neighborhood-level price of housing reduced all-cause mortality by 5% after adjusting neighborhood-level factors.
• Housing prices and educational composition were related and explained part of the variance of all-cause mortality.

## 1 Introduction

In a society where housing is highly commercialized, the residence is mostly determined by people's ability to mobilize their socioeconomic resources (Bernard et al., 2007). Housing is a unique asset that generates revenue in the form of vested rent and provides housing services (Atkinson, 2015; Piketty, 2014). Housing systems, such as housing policies and the real estate market, and the social and physical environment jointly influence housing values (Mari-Dell'Olmo et al., 2017; Mehdipanah et al., 2017). Thus, housing value reflects neighborhood and individual-level conditions, including neighborhood social status, wealth, and housing conditions (Mehdipanah et al., 2017).

In many cities, economic inequality in urban areas increases (Massey, 1996; OECD, 2018; World Health Organization & United Nations. Human Settlements Programme., 2010). Slater asserted that problems that arise in cities turn into a product of capital accumulation and class conflict when the assumption is converted from “where you live affects your life” to the belief that “your life affects where you live.” (Slater, 2013) Bambra and colleagues also argued that the question “where is power and who works?” should be asked (Bambra et al., 2019). Another study also stated that urban health inequalities stemmed from “systematically unequal distribution in power, prestige, and resources associated with relative position in the social hierarchy.” (Friel et al., 2011) Taking the housing price into account would make it possible to examine cities’ health problems through the lens of health inequality.

Korea is one of the countries that has experienced rapid economic growth since the 1960s (Deaton, 2015). The capital Seoul has rapidly become urbanized. London developed from one million to eight million residents in approximately 130 years, while Bangkok took 45 years, and Seoul only 25 years (World Health Organization & United Nations. Human Settlements Programme., 2010). The state's role was to promote industrialization by mobilizing political and economic means (D. Kim, 2018). Under the development ideology of the industrialization period, the authoritarian government united with economic power, while civil society and laborers were excluded from policy paradigms formation and development profit distribution (Hwang, 2016). Survival through diligent household saving was prioritized over public welfare policies. The housing provision policy was implemented to alleviate the distribution conflict and grow the middle class, which has been friendly to the ruling powers. The fruits of economic growth were realized individually through housing market gains using savings as leverage (H. Park, 2013).

Korea's housing system is highly market-driven, and housing accounts for a substantial portion of capital. As of 2019, real estate accounts for 75.5% of total wealth, which is much higher than other countries, of which housing accounted for 55.0% (Statistics Korea, 2019). High housing values are clustered in Seoul's downtown area, especially in the southeastern part of the city (Gangnam area) and its surroundings (B. Park & Jang, 2016). It contrasts with the United States and some European countries, where central urban districts are economically deprived and have been subject to gentrification in general (Galea et al., 2019). These areas attracted middle-class residents due to their dense upmarket physical and social environments, including excellent educational opportunities, convenient transportation, abundant consumption and cultural facilities, and stable housing asset values (E. Choi, 2004, 2006; B. Park & Jang, 2016).

The spatial distribution and changes in the neighborhood and individual socioeconomic position (SEP) can interactively cause intra-urban variation in health levels (Cummins et al., 2007; A. V. Diez Roux et al., 2007; Friel et al., 2011; Galea et al., 2005; Meijer et al., 2012; Northridge et al., 2003). Recent Korean studies argued that housing prices were among the most important factors for internal migration (H. Y. Lee & Lee, 2008; Song & Kim, 2016). A study found that the internal migration pattern formed a gradient by SEP because housing price influences residential mobility (C. Kim, 2015). Therefore, the magnitude of association between housing prices and all-cause mortality needs to be estimated better to understand the spatial patterning of mortality in Seoul. Understanding Seoul's processes also would help understand the health inequality of the country's urban spaces because Korea's other regions have mimicked what has happened in the capital (Hwang, 2016).

### 1.1 Aim of study

This study assessed the ecological association between neighborhood-level all-cause mortality and housing value. First, we explored the spatial distribution of housing prices and all-cause mortality in Seoul. Second, we estimated the housing price elasticity of all-cause mortality using a pooled ordinary least squares (OLS) model. The educational composition was added to the model to explore the possible mediating role of housing prices in the pathway of educational composition on all-cause mortality. We defined neighborhoods’ educational composition as a proportion of people who completed primary, secondary, and tertiary education in each neighborhood. Third, a spatial fixed-effects regression analysis was performed, and the magnitudes of elasticity were compared with those from the pooled OLS model.

## 2 Material and methods

### 2.1 Data

The six years of the entire study period (2013–2018) were divided into three discrete periods (2013–2014, 2015–2016, 2017–2018). The standard housing rental contract period in Korea is two years, so we assumed that population movement would mainly occur in two years. It is also known that if the relative risk is between 1.5 and 2.0, the empirical Bayes is not powerful when the denominator does not exceed 70 (Ugarte et al., 2009). When the two-year data were combined, expected deaths were less than 70 in 3.2% of all neighborhoods.

To estimate neighborhood-level mortality, we first acquired the number of populations and deaths by neighborhood from the Ministry of Interior and Safety. The neighborhood-level annual population and death counts have been collected and published online (https://www.mois.go.kr/). Second, we calculated the mid-term population by period/gender/neighborhood using the number of populations at the end of December every year.

For each neighborhood's housing price, we used the housing actual price data of the Ministry of Land, Infrastructure, and Transport, which contains the country's entire real housing transaction price. In Korea, by law, all house sales contract information must be reported to the authority within one month of the contract's date (Ministry of Land Infrastructure and Transport, 2020). The authority discloses the collected data through its website, and the disclosed information includes address, sale price, and contract date (https://rt.molit.go.kr/).

The Seoul Metropolitan Government's information disclosure system (Seoul Open Data Plaza, http://data.seoul.go.kr) was used to acquire each neighborhood's characteristics. Educational composition data were obtained from the 20% sample of 2015 Census data.

### 2.2 Unit of analysis

The smallest administrative unit was set as the unit of analysis, the smallest unit to obtain the data used herein. As of Dec 31, 2018, 424 units were adjusted to 423 in consideration of administrative change.

### 2.3 Outcome variable

All-cause mortality was used as the outcome variable. All-cause mortality for each neighborhood was estimated using the gender-age-adjusted standardized mortality ratio (SMR).

### 2.4 Variable of interest

The variable of interest was the median value of housing prices in neighborhoods. We assumed the median housing price as an appropriate proxy for the local area's SEP (Oakes & Andrade, 2017; U.S. Census Bureau, 2014). During the entire study period, there were a total of 928,405 housing transactions in Seoul. Geographic coordinates were derived using the contract address. The smallest administrative unit's centroid designated the coordinates in 19,348 (2.1%) cases where the exact address was unclear.

### 2.5 Explanatory variables

#### 2.5.1 Selection of variables

Previous studies have suggested that physical and social environments, population composition, health, and social services as urban living conditions that influence health outcomes (Borrell et al., 2013; A. V. Diez Roux & Mair, 2010; Friel et al., 2011; Galea et al., 2005; Macintyre et al., 2002; Vlahov et al., 2007). The physical environment includes transportation systems, housing, population density, pollution, and the social environment includes economic development, quality of education, social norms, and social capital (Borrell et al., 2013; A. V.; Diez Roux & Mair, 2010; Galea et al., 2005; Northridge et al., 2003). We used four neighborhood-level factors (poverty rate, population density, business workers density, the number of nearby subway stations), and four district-level factors (the number of physicians per 1000 population, particulate matter (PM) 10 annual concentration, park area per person, and local tax amount). We also used neighborhood-level educational composition for exploring the possible mediating role of housing prices on the educational composition's effect on all-cause mortality. A path diagram for the models used in this study is shown in Fig.A.1.

#### 2.5.2 Neighborhood and district-level variables

We estimated the poverty rate using the proportion of Basic Livelihood Security Program (BLSP) benefits recipients. We included the poverty rate as an explanatory variable to supplement primary education proportion for capturing the current SEP fully. The Pearson correlation coefficient between the poverty rate and housing prices was −0.570. The Pearson correlation coefficients between the poverty rate in 2015 and primary, secondary, and tertiary education were 0.582, 0.472, and −0.508. The Korean government pays BLSP benefits to households below a certain standard, considering income, poverty, and dependent family size. As of 2018, the proportion of BLSP recipients is about 3.4% of the national population. In previous Korean studies, the proportion of BLSP beneficiaries was also used as a proxy for poverty (Y. A. Lee, 2015; Y. Park, 2011) because it is difficult to obtain the poverty level of neighborhoods directly. The density of population and business workers were estimated using each neighborhood area (km^2^) as the denominator. The nearby subway station was set as the number of subway stations within a 10-min walk. Educational composition and poverty rates were adjusted for gender and age using an indirect standardization method in the same way as the outcome variable (Milyo & Mellor, 2003). PM10 is the mean annual district-level PM10 concentration in μg/m^3^. Local tax amounts were associated with the district's SEP and financial independence from the Central government (Bae & Lee, 2008; Yoo & Han, 2007).

Multicollinearity for our model was checked. When we calculated magnitudes of the correlation among explanatory variables, all the Pearson correlation coefficient magnitudes were under 0.8–0.9, which is considered the threshold at which multicollinearity is possibly present (Midi et al., 2013). One variable, local tax amount, had 2.7 of variance inflation factors (VIF). However, the tolerance value (0.37) was over 0.3, considered one of the thresholds for a potential collinearity problem (Midi et al., 2013). In addition, remove of local tax amount moved the housing prices’ effect slightly away from the null. We assumed that the model is free of collider bias with no or negligible effect of SMR on explanatory variables (Luque-Fernandez et al., 2019).

### 2.6 Statistical analysis

#### 2.6.1 Estimation of the spatial distribution of all-cause mortality

An indirect standardization method was applied to calculate the all-cause mortality as a gender-age-adjusted SMR. The standard population was the total population of Seoul from 2015 to 2016. Each neighborhood's SMR was smoothed using the empirical Bayes method. If the main goal is smoothing the relative risk, the empirical Bayes method yields result similar to the full Bayes method (Ugarte et al., 2009). We mapped the distribution of neighborhood-level housing prices and all-cause mortality in Seoul during the study period. Global and local Moran's I value (Anseline, 1995) were used to examine the degree of spatial clustering of the two variables.

#### 2.6.2 Estimation of housing prices elasticity of all-cause mortality using a pooled OLS model

The educational composition could only be obtained from the 2015 census data. We, therefore, performed a pooled OLS analysis using education-level variables. We regressed unsmoothed SMR on housing prices and other explanatory variables. All variables were log-transformed to form a log-log model. The equation used in the pooled OLS analysis was as follows:

$$\text{ln}\left(\mu_{i}\right)=\underset{j}{\sum }\text{ln}\left(X_{ji}\right)\beta_{j}+u_{i}$$

where $\mu_{i}$ is the unsmoothed SMR of neighborhood *i*, $X_{ji}$ is the *j*th explanatory variable of neighborhood *i*, $\beta_{j}$ is the regression coefficient of the *j*th explanatory variable, and $u_{i}$ is an error term of neighborhood *i*.

A simple and robust Lagrange multiplier (LM) test using non-spatial regression models was performed to examine the existence of spatial autocorrelation (Anseline et al., 1996). We also estimated the Global and Local Moran's I values of the residuals of the non-spatial Poisson model.

#### 2.6.3 Spatial fixed-effects regression analysis

Previous studies have attempted to solve the spatial autocorrelation problem by including additional variables in the regression equation (Franzese & Hays, 2008; Ross & Homer, 1976). We organized data into time-series cross-sectional data and analyzed it using space and time fixed-effects spatial Durbin error model (SDEM). SDEM is a combination of the spatially lagged-X (SLX) model that assumes the effect of the explanatory variables of the neighboring areas on the outcome variable of the area (spillover effect), and the spatial error model (SEM), which assumes the interaction between the unobserved variables in neighboring areas and the area (Elhorst, 2017; LeSage, 2014; Vega & Elhorst, 2015). We believe that the local spillover effect was more appropriate as the outcome variable of the current study was a social aggregate (Cook et al., 2020; Franzese & Hay, 2016, pp. 83–87). We constructed the row-standardized queen contiguity spatial weights of order 1. R^2^ and pseudo-R^2^ values were used to determine the fitness of the models. The equation used in the spatial panel analysis was as follows:

$$\text{ln}\left(\mu_{it}\right)=\underset{j}{\sum }\text{ln}\left(X_{jit}\right)\beta_{j}+\underset{j}{\sum }W\times \text{ln}\left(X_{jit}\right)\theta_{j}+\alpha_{i}+\gamma_{t}+u_{it}$$

$$u_{it}=\lambda Wu_{it}+\varepsilon_{it}$$

where $\mu_{it}$ is the unsmoothed SMR of neighborhood *i* at time *t*, $X_{jit}$ is the *j*th explanatory variable of neighborhood *i* at time *t*, *W* is the contiguity weight matrix, $\theta_{j}$ is the regression coefficient of the *j*th spatially lagged explanatory variable. $\alpha_{i}$ is the fixed effects of neighborhood *i*, and $\gamma_{t}$ is the fixed effects of time. $u_{it}$ is an error term of neighborhood *i* at time *t*, composed of spatial and non-spatial factors ($\varepsilon_{it}$). λ is the coefficient value for the spatial error term.

This study used the “splm” package in R 3.6.3 (https://www.r-project.org) and QGIS 3.12.1 (https://www.qgis.org), GeoDa 1.14.0 (https://geodacenter.github.io) for the analysis.

## 3 Results

### 3.1 Characteristics of neighborhoods

The characteristics of neighborhoods by period are reported in Table 1. The median population in 2013–2014 was 24,108, the minimum value was 1495.5, and the maximum value was 673,374.5. The interquartile range (IQR) was 12,879.8. The median number of deaths in 2013–2014 was 185, and the minimum and maximum were 8 and 492, respectively. The median population tended to decrease, but the median number of deaths increased. In 2013–2014, the median housing price was 366.6 million KRW (IQR 171.5 million KRW, 1 million KRW is approximately 900 US dollars as of Nov. 2020). The minimum was 135.5 million KRW, and the maximum was 1480 million KRW. The median housing price increased by 13.3% in 2015–2016 compared to 2013–2014 and 21.4% in 2016–2017 compared to 2014–2015. The IQR also rose significantly from 171.5 in 2013–2014 to 301.0 in 2017–2018. This difference seems mainly due to the rise in prices in neighborhoods where housing prices were already high (Fig.A.2). Compared to each decile's value in 2013–2014, the rate of increase was greater in the high housing price decile (A). In comparing the boundary value for each decile and the median value (50 percentile), the higher decile boundary value increased more (B).

**Table 1:** Characteristics of study subjects by period.

Variables	2013–2014 (N = 423)		2015–2016 (N = 423)		2017–2018 (N = 423)	
	Median(Min, Max)	IQR	Median(Min, Max)	IQR	Median(Min, Max)	IQR
Neighborhood-level						
Population, n	24108 (1495.5, 673374.5)	12879.8	23706.0 (943.0, 666213.0)	12312.5	23613.0 (1010.0, 672340.0)	12863.2
Death, n	185 (8, 492)	96	192 (10, 497)	113	194 (12, 573)	118
Housing price, 1 million KRW	366.6 (135.5, 1480.0)	171.5	415.5 (140.0, 1530.0)	236.0	504.6 (135.0, 2565.0)	301.0
Poverty	0.71 (0.01, 6.19)	0.51	0.93 (0.01, 6.42)	0.70	0.99 (0.01, 6.04)	0.73
Population density, inhabitants/km^2^	24782.8 (742.7, 65563.0)	17927.3	24329.1 (703.7, 63438.6)	17426.2	24071.6 (753.7, 60116.7)	17783.3
Business workers, employees/km^2^	6169.85 (318.8, 105533.3)	7048.5	6813.3 (341.0, 120243.9)	7641.2	6859.7 (391.6, 110341.9)	7188.8
Subway stations, n	3 (1, 17)	4	3 (1, 17)	4	4 (1,17)	4
Primary education			0.98 (0.13,1.99)	0.42		
Secondary education			0.97 (0.13,1.52)	0.36		
Tertiary education			0.95 (0.35,1.82)	0.36		
District-level						
Doctors, no. of doctors/1000 inhabitants	2.1 (0.8, 12.3)	2.7	2.5 (1.0, 12.3)	2.6	2.2 (1.0, 13.9)	2.3
PM10 concentration, μg/m^3^	45.5 (42.0, 48.5)	2.5	47.0 (42.0, 52.0)	3.0	42.5 (36.0, 48.5)	3.8
Park area, m^2^ per se	11.0 (3.4, 69.5)	15.7	11.2 (3.4, 71.0)	16	11.5 (3.5, 70.7)	15.8
Total amount of local tax, 1 million KRW	343204.0 (175219.5, 2064262.5)	386485	417424.0 (211867.0, 2862131.5)	403339.5	477003.5 (229249.0, 3014322.0)	490568.5

*Notes.* IQR = Interquartile range; KRW = Korean Won; Max = Maximum; Min = Minimum; PM = Particulate matter.

### 3.2 Spatial distribution of SMR

**Fig. 1:**
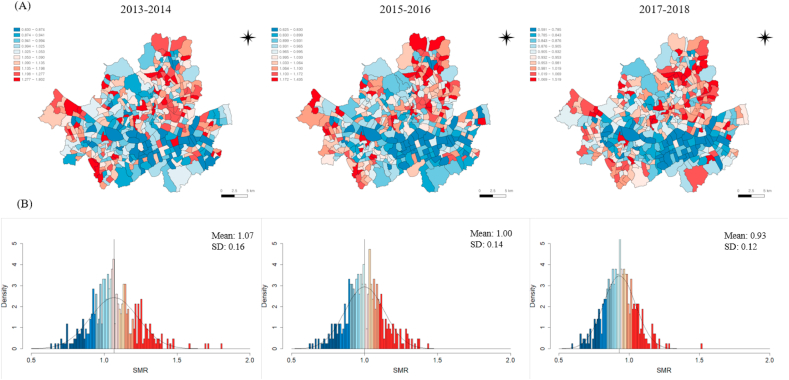
Mapped distribution (A) and histogram (B) of SMR deciles in Seoul during the study period. *Notes.* The solid black line in the lower panel indicates the normal distribution using the mean and SD of the SMR. SD = Standard deviation; SMR = Standardized mortality ratio.

The mapped distribution and histogram of smoothed SMR in Seoul during the study period are depicted in Fig. 1. In 2013, the mean value of the smoothed SMR was 1.07, and the standard deviation (SD) was 0.16, all of which decreased with time. The maximum and minimum SMR value was 2.86 in 2013–2014, 2.30 in 2015–2016, and 2.57 in 2017–2018. During the entire study period, the southeastern part of Seoul and its surroundings showed a relatively lower SMR than the other parts of Seoul. On the other hand, the northern and some western parts clustered higher SMR than other areas.

The ratio of each decile value of SMR in 2013–2014 to each decile in 2015–2016 and 2017–2018 is presented in Fig.A.3. All ratios decreased with time, especially in the high mortality deciles (A). The ratio of the SMR for each decile to the median value was also shown by period (B). The ratio between the 10–40 percentiles and the median value did not change significantly with time. However, the ratio decreased substantially in the 60–90 percentiles and median.

### 3.3 Spatial distribution of housing prices and educational composition

**Fig. 2:**
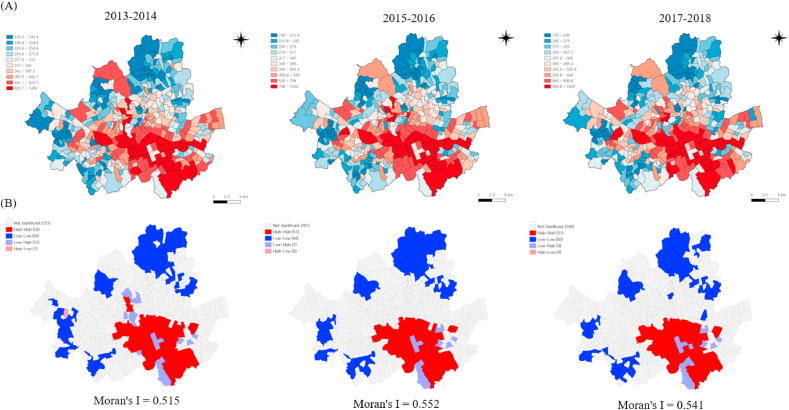
Mapped distribution (A) and spatial patterning (B) of neighborhood-level housing prices in Seoul by study period. *Notes*. Housing prices in 1 million KRW.

The spatial median housing price distribution for each period is mapped in Fig. 2. Housing prices were high in the southeastern part and its surroundings, and low housing prices were observed in Seoul's northern and some western parts. The degree of clustering measured by Global Moran's I increased (0.515 in 2013–2014 and 0.541 in 2017–2018). The marginal and joint distribution of log-transformed housing prices and SMR for each period are shown in Fig.A.4. SMR and housing prices showed a negative correlation. The Pearson correlation coefficients (r) were the smallest at −0.456 in 2013–2014 and the largest at −0.538 in 2015–2016.

Each neighborhood's educational composition in 2015 is mapped and the Global and Local Moran's I at each educational level is estimated in Fig.A.5. We found a similar spatial pattern between tertiary education proportion and housing price. We also found mirror images of primary and tertiary education. Global Moran's I values were 0.557 in primary education, 0.614 in secondary education, and 0.572 in tertiary education. Pearson correlation coefficients among neighborhood-level educational composition and housing price are shown in Table A.1. Primary and secondary education variables showed a significant negative association with the median housing price variable (−0.690 in primary education, −0.794 in secondary education). In contrast, the tertiary education variable showed a positive association (r = 0.741).

### 3.4 Housing prices elasticity of all-cause mortality

The results of Global and Local Moran's I values for the z-transformed residuals of SMR estimated using the Poisson regression model are presented in Fig.A.6. Compared to 2013–2014, Global Moran's I value increased in 2015–2016 and 2017–2018 (0.170 in 2013–2014, 0.269 in 2015–2016, and 0.258 in 2017–2018). In the LM test, spatial autocorrelations were significant in all-cause mortality (Table A.2.). However, significant spatial autocorrelation disappeared after the neighborhood's educational composition was added to the model (Model 4) in 2015–2016.

The results of the pooled OLS regression analysis are reported in Table 2. In Model 1, the 1% increase in housing price was associated with a 0.20% decrease in all-cause mortality (95% confidence interval (CI) −0.22 to −0.18). Models 2 and 3 attenuated the regression coefficient after adding the other neighborhood and district-level explanatory variables but were still significantly associated with the mortality decrease. When the educational composition variable was added to the model (Model 4), the magnitude of regression coefficient of the housing price variable was −0.05 (95% CI -0.08 to − −0.03).

**Table 2:** Housing price elasticity of all-cause mortality: Results of a pooled OLS regression analysis.

Variable	Model 1		Model 2		Model 3		Model 4	
	Coefficient (95% CI)	P-value	Coefficient (95% CI)	P-value	Coefficient (95% CI)	P-value	Coefficient (95% CI)	P-value
Intercept	1.17 (1.07,1.28)	<0.001	1.30 (1.12,1.48)	<0.001	0.83 (0.34,1.32)	<0.001	−0.06 (−0.57,0.44)	0.803
Neighborhood-level								
ln(Housing price)	−0.20 (−0.22,-0.18)	<0.001	−0.15 (−0.17,-0.13)	<0.001	−0.11 (−0.13,-0.08)	<0.001	−0.05 (−0.08,-0.03)	<0.001
ln(Poverty)			0.07 (0.06,0.07)	<0.001	0.07 (0.06,0.08)	<0.001	0.02 (0.01,0.04)	0.002
ln(Population density)			−0.03 (−0.04,-0.01)	<0.001	−0.04 (−0.05,-0.02)	<0.001	−0.04 (−0.05,-0.03)	<0.001
ln(Business workers)			0.01 (0.00,0.02)	0.057	0.02 (0.01,0.03)	<0.001	0.02 (0.01,0.03)	<0.001
ln(Subway stations)			0.01 (0.00,0.02)	0.162	0.01 (−0.01,0.02)	0.280	0.00 (−0.01,0.01)	0.855
ln(Primary education)							0.10 (0.06,0.14)	<0.001
ln(Tertiary education)							−0.15 (−0.20,-0.10)	<0.001
District-level								
ln(Doctors)					0.03 (0.01,0.05)	<0.001	0.02 (0.01,0.04)	0.003
ln(PM10 concentration)					0.32 (0.21,0.43)	<0.001	0.33 (0.22,0.44)	<0.001
ln(Park area)					−0.02 (−0.03,-0.01)	0.001	−0.01 (−0.02,0.01)	0.276
ln(Local tax)					−0.07 (−0.09,-0.05)	<0.001	−0.04 (−0.06,-0.02)	<0.001
R-squared	0.283		0.381		0.419		0.463	

*Notes.* Model 1 includes the intercept and housing prices in the model. In Model 2, we added the neighborhood-level variables (poverty rate, population density, business workers density, the number of nearby subway stations) to Model 1. In Model 3, we further added the district-level variables (the number of physicians per 1000 population, PM10 annual concentration, park area per person, local tax) to Model 2. In Model 4, the educational composition was added to Model 3.

CI = Confidence interval; PM = Particulate matter.

The results of the spatial fixed-effects regression analysis are reported in Table 3. In Model 5, the regression coefficient for housing prices was −0.05 (95% CI -0.07 to −0.02), in which the magnitude was similar to the results in Model 4. The magnitude of the regression coefficients in poverty and population density was relatively large and significant. Among the spatially lagged variables, the housing price and subway station variables showed a statistically significant association. In Model 6, we added district-level explanatory variables and found that the magnitude and direction of the association were not significantly changed.

**Table 3:** Housing price elasticity of all-cause mortality: Results of a spatial panel analysis.

Variable	Model 5		Model 6	
	Coefficient (95% CI)	P-value	Coefficient (95% CI)	P-value
Neighborhood-level				
ln(Housing price)	−0.05 (−0.07,-0.02)	<.001	−0.04 (−0.07,-0.02)	0.001
ln(Poverty)	0.09 (0.08,0.10)	<.001	0.09 (0.08,0.10)	<.001
ln(Population density)	−0.05 (−0.06,-0.03)	<.001	−0.05 (−0.06,-0.04)	<.001
ln(Business workers)	0.02 (0.00,0.03)	0.005	0.02 (0.01,0.03)	0.001
ln(Subway stations)	−0.02 (−0.04,0.00)	0.013	−0.03 (−0.04,-0.01)	0.005
W x ln(Housing price)	−0.05 (−0.09,-0.01)	0.012	−0.04 (−0.08,0.00)	0.074
W x ln(Poverty)	0.02 (0.00,0.04)	<.001	0.02 (0.00,0.04)	0.036
W x ln(Population density)	0.06 (0.04,0.09)	0.120	0.05 (0.03,0.08)	<.001
W x ln(Business workers)	−0.01 (−0.03,0.01)	0.505	0.00 (−0.02,0.02)	0.890
W x ln(Subway stations)	0.04 (0.02,0.07)	0.002	0.04 (0.01,0.07)	0.005
District-level				
Doctors			0.01 (−0.01,0.03)	0.286
PM10 concentration			−0.04 (−0.18,0.10)	0.544
Park area			−0.02 (−0.03,0.00)	0.006
Local tax			−0.03 (−0.05,-0.01)	0.007
Pseudo R-squared	0.676		0.679	

*Notes.* We adjusted the neighborhood-level variables (poverty rate, population density, business workers density, and the number of nearby subway stations) and the mean values of adjacent neighborhoods in Model 5. We further adjusted the district-level variables (the number of physicians per 1000 population, PM10 annual concentration, park area per person, and local tax) in Model 6.

CI = Confidence interval; PM = Particulate matter; W = Spatial weight matrix.

## 4 Discussion

### 4.1 Summary of findings

Calculating the smoothed SMR for each neighborhood in Seoul revealed that the mean and the variance decreased over time, but the spatial patterning increased. Housing prices were also spatially more clustered. Using the pooled OLS model, the all-cause mortality decreased by approximately 0.11% as the housing price increased by 1%. After adding the educational composition to the model, the magnitude of the housing price effect was attenuated (a 1% increase in housing prices was related to a 0.05% mortality decrease). The spatial autocorrelation was not statistically significant when including educational composition and housing prices simultaneously in the model. When we used the fixed-effects SDEM, a 1% increase in housing prices was related to a 0.05% decrease in all-cause mortality.

### 4.2 Advantages of housing prices as neighborhood-level SEP

The use of a neighborhood-level SEP has several advantages. It can be used for all ages and is similarly applied to gender (Krieger et al., 1997). It is also relatively more stable than income or job indicators, mainly reflecting current circumstances (Krieger et al., 1997; Pickett & Pearl, 2001). It is also possible to include those excluded when using traditional occupational-based indicators (Seaman et al., 2019). A single variable, housing prices closely related to the level of wealth, was used to proxy the neighborhood-level SEP. Wealth is regarded as a source of economic security and power and can function as a buffer against economic shocks (Krieger et al., 1997). A single variable measure is relatively easy to calculate, and it can be estimated every year to ensure continuity. Previous studies conducted in Korea mainly used the deprivation index as a regional SEP indicator (M. H. Choi et al., 2011; Shin et al., 2009; Yoon et al., 2015). However, if a single index that can explain the specific association is used, the process of determining the urban health level would be better understood.

### 4.3 Changes in mean and variance of neighborhood-level SMR

The mean and variance of all-cause mortality were found to decrease simultaneously. In particular, the forces to lower mortality in areas with high mortality have reduced dispersion. Two recent studies presented mixed results in changes in the variance of all-cause mortality. A study conducted in Germany observed time-series changes in German state-level mortality during a 25-year study period and found that mortality levels converged despite large economic gaps between regions (van Raalte et al., 2020). On the other hand, a study conducted in Scotland revealed that lifespan variation increased during 1981–2011 (Seaman et al., 2019). The first reason for the decrease in mortality variation could be the weaker association between housing prices and mortality as the housing prices increase. Preston suggested the Preston curve, which demonstrated a log-linear relationship between regional gross domestic product (GDP) per capita and life expectancy (Preston, 1975). As of 2017, the mean life expectancy in Seoul was 82.57 years, 80.18 years for males, and 85.01 years for females. In the situation where the declining force of all-cause mortality was saturated, it is possible that the mortality in areas where the SEP was relatively high and the mortality was low stagnated, whereas the mortality decreased where the SEP was low. Another possible cause is residential mobility. The housing market plays a key role in determining where urban residents live (Modai-Snir & van Ham, 2018; Smith & Easterlow, 2005). In particular, when housing prices rise rapidly, residents with low SEP are less likely to afford to purchase or rent houses, and they can be excluded from those areas involuntarily (Modai-Snir & van Ham, 2018). Seoul has a much higher housing price and living cost than other regions. In addition, the rate of increase in housing prices has been steeper in Seoul than in other regions. In 2010, real estate assets were a significant movement indicator outside Seoul (Ryu et al., 2013). Therefore, we speculate that the movement of low SEP outside Seoul as housing price rises could induce a decrease in mortality variation. The application of social and public health policies or health systems can be another potential cause (Asaria et al., 2012).

### 4.4 Spatial patterning of housing prices and all-cause mortality

According to the current study results, the spatial patterning of housing prices and all-cause mortality increased, especially housing prices. Kramer argued that socioeconomic resource distribution was one of the most important causes of spatial health inequality (Kramer, 2016). Neighborhood differences are not naturally formed but are shaped through various social and economic processes (A. DiezRoux, 2002; Link, 2008). Since the late 1960s, urbanization in Korea has progressed along with industrialization led by the central government. Rapid urbanization leads to an increase in the overall economic level, creating socioeconomic inequalities within the city (Friel et al., 2011). The development of the southeastern part of Seoul began in the 1960s, and the middle class rushed into the area. The changes in the proportion of those who received tertiary education among the district's population on a 10-year basis are shown in Fig.A.7. Between 1975 and 1985, the proportion of tertiary education in the southeastern part of Seoul, indicated in red, increased sharply. This rate solidified between 1985 and 1995. The rich educational and cultural environments that were constructed to promote the internal migration of the middle class at the time of development brought educational capital inheritance to the next generation and a stable housing asset value (B. Park & Jang, 2016). As affluence became visible, the southeastern part of Seoul became the object of desire and was imitated across the country (Hwang, 2016). Some previous studies argued that the composition and contextual effect division is a false dualism, and they mutually reinforce each other (Bambra et al., 2019; Frohlich et al., 2001; Meijer et al., 2012). Thus, spatial clustering potentially solidifies over time and affects residents' health by itself (A. V. Diez Roux & Mair, 2010; Krieger et al., 2018).

### 4.5 Association between housing prices and SMR

The final pooled OLS and spatial panel model commonly found that a 1% increase in housing prices lowered mortality by approximately 0.05%. After adding the educational composition to the pooled OLS analysis, the coefficient of housing prices attenuated. These results suggest that housing prices and educational composition were related and explained part of the variance of all-cause mortality. Access to health-protecting resources, such as health care, better nutrition, and hygiene, seems to be determined depending on education level (Jerrett et al., 1998; Rydland et al., 2020). There are also differences in health-related knowledge, health behaviors, and attitudes to potential health threats (Osler & Prescott, 2003). Fundamental cause theory explains that differences in health outcomes arise due to relative differences in access to resources according to the social hierarchy (Link & Phelan, 1995; Phelan et al., 2010). Kim and colleagues found that the residences of Seoul's power elites were clustered in the city's southeastern area (C. S. Kim et al., 2006). Considering the evident health inequality according to the SEP in Korea (Jung-Choi et al., 2011; Khang et al., 2004), Seoul's spatial health inequalities seem to appear through the high SEP population's affinity for a better environment sorted by housing price. In addition to housing prices, variables related to place development or composition changes, such as poverty rate or population density, significantly influenced the mortality rate. A previous study revealed that a higher population density lowered the region's health levels (Vlahov & Galea, 2002). However, in Seoul, the supply of large residential complexes, such as high-rise apartments, has been closely related to areal development. This raises the importance of area development and population composition change in determining the spatial patterning of mortality levels.

### 4.6 Limitations

The current study has some limitations. First, additional variables may affect mortality. For example, the region's informal reciprocity level affects health, but this variable could not be included in the study. We utilized a fixed-effect spatial panel analysis to minimize the omitted variable bias. Second, this study adopted an ecological design using time-series cross-sectional data. The disadvantage of an ecological study is that it is impossible to partition the influence of factors at various levels. Merlo et al. also emphasized the importance of individual-level factors in small-area studies (Merlo et al., 2012). However, the purpose of this study was not to sharply separate the effect of contextual and compositional factors. Rather, it was to clarify neighborhood-level mortality and SEP's spatial distribution and their association under the implicit assumption that compositional and contextual factors reinforce each other. It should be noted that the results should be explained at the group level. Third, the change in population composition according to neighborhood SEP changes could not be captured due to data limitations. Housing prices were one of the major factors that affected the internal migration pattern of Seoul and the capital area (C. Kim, 2015; H. Y. Lee & Lee, 2008; Min & Byun, 2017). Residential mobility affects the socioeconomic and demographic characteristics, including health outcomes, of the space promptly. Thus, internal migration should be accounted for spatial mortality variation. Fourth, a neighborhood was used as the smallest administrative unit. Setting an administrative unit as such may differ from the residents' perception or actual living circumstances. However, using an administrative unit can aid policy decisions or resource allocation (Krieger et al., 2018). Furthermore, the effect of various neighborhood definitions was not substantively significant on health inequality estimates (Stafford et al., 2008). Fifth, actual transaction data were used to estimate the median housing price of the neighborhood. It has the advantage of being able to measure better the actual value of the house rather than the appraisal data. However, since it only includes the housing in which the transaction was made, it can over- or under-represent a specific type of housing.

In future studies, quasi-experimental research designs should be employed to reveal the urban space health inequality mechanism—changes in housing prices, population composition, and health levels with exogenous events. Their associations would clarify some aspects of the mechanism. In addition, given the strengthened spatial patterning of all-cause mortality and housing prices, we consider spatial patterning effects on health levels as one of the important themes of future studies.

## 5 Conclusions

This study explored the spatial distribution of all-cause mortality and housing prices and estimated the elasticity of housing prices to all-cause mortality. There were differences in change patterns in these, but an increase in spatial patterning was common. The association between housing prices and all-cause mortality was significant. When the educational composition and housing prices were added to the model simultaneously, spatial autocorrelation was not significant, and the effect of housing prices attenuated, indicating its potential mediating role in the educational composition's effect on all-cause mortality. This study may provide additional insights into potential urban space health inequality mechanisms.

## Financial disclosure statement

This research did not receive any specific grant from funding agencies in the public, commercial, or not-for-profit sectors.

## Declaration of competing interest

None.

## Author statement

KIH has sole responsibility for the following: study conception and design, data collection, analysis and interpretation of results, and manuscript preparation.
