# Supplementary material for: Spatial distribution of neighborhood-level housing prices and its association with all-cause mortality in Seoul, Korea (2013–2018): A spatial panel data analysis
Source: SSM Popul Health. 2021 Nov 11;16:100963. doi: 10.1016/j.ssmph.2021.100963 (PMC8599165; doi:10.1016/j.ssmph.2021.100963)
Supplement: Multimedia component 1 [file mmc1.docx]

**Appendices**


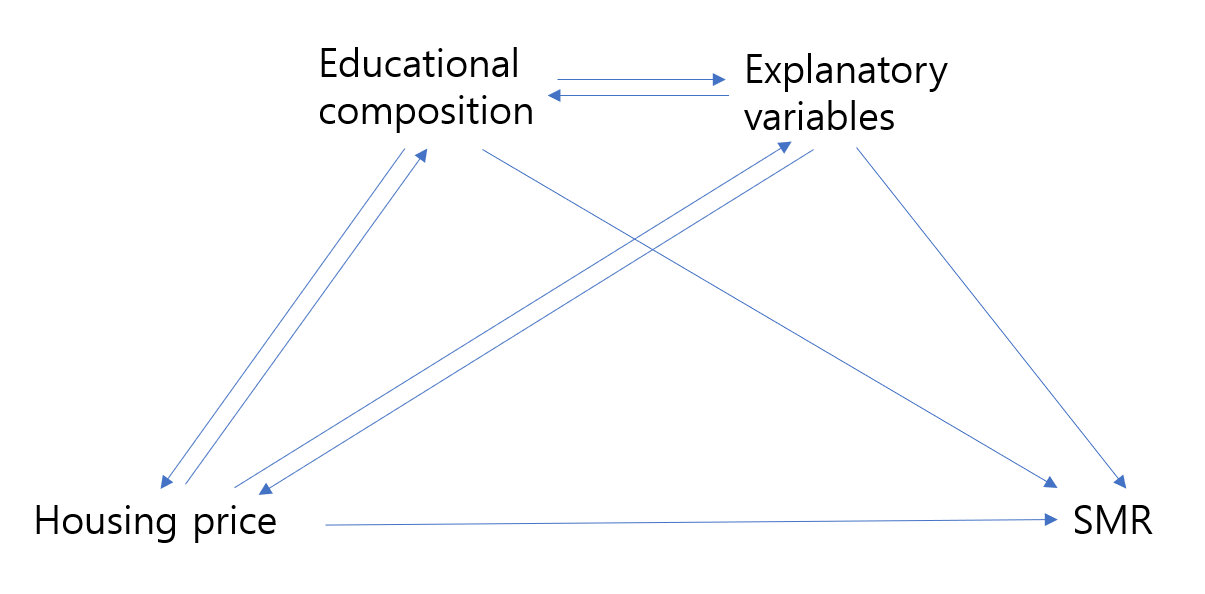


Fig.A.1. Path diagram for the study model.

*Notes.* SMR = Standardized mortality ratio.


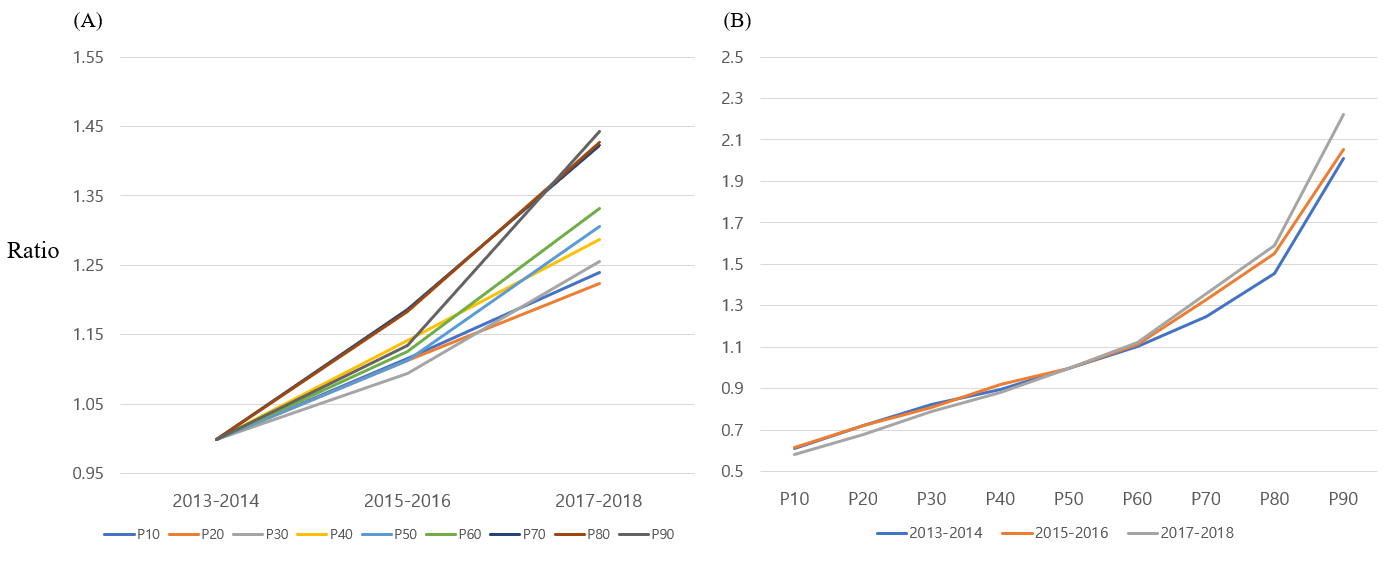


Fig. A.2. Changes in the boundary value for each decile of housing prices during the study period. (A) The ratio of the boundary values for each decile of the housing prices in 2015–2016, 2017–2018 compared to 2013–2014. (B) The ratio between the deciles of the housing prices to the median for each period.


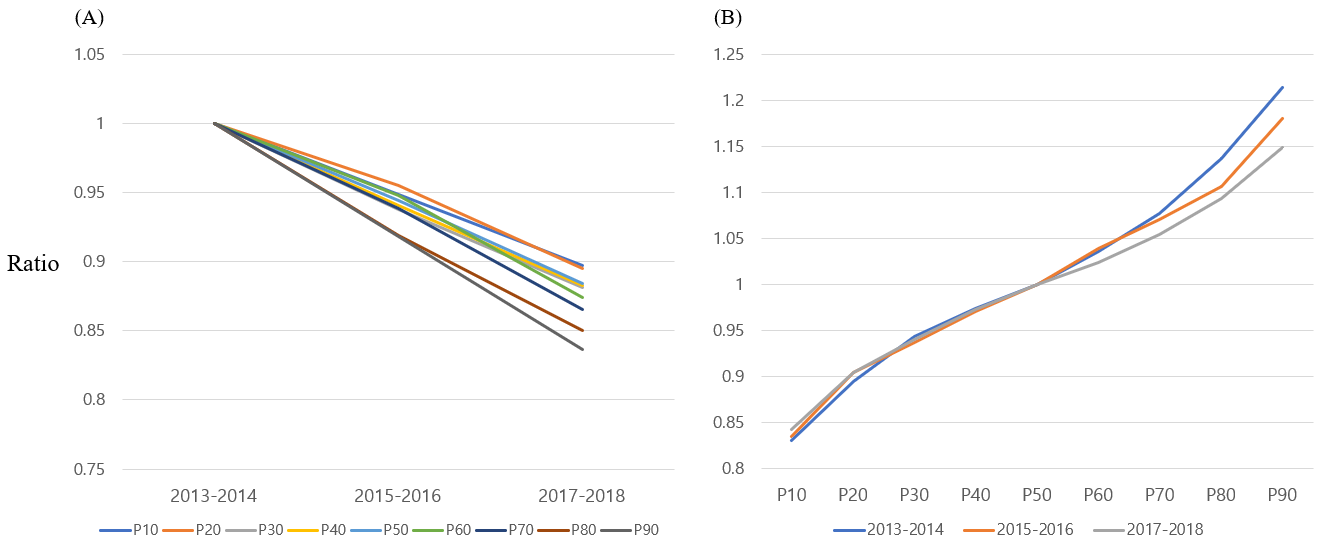


Fig. A.3. Changes in the boundary value for each decile of SMR during the study period. (A) The ratio of the boundary values for each decile of the SMR in 2015–2016, 2017–2018 compared to 2013–2014. (B) The ratio between the deciles of the SMR to the median for each period.

*Notes.* SMR = Standardized mortality ratio.


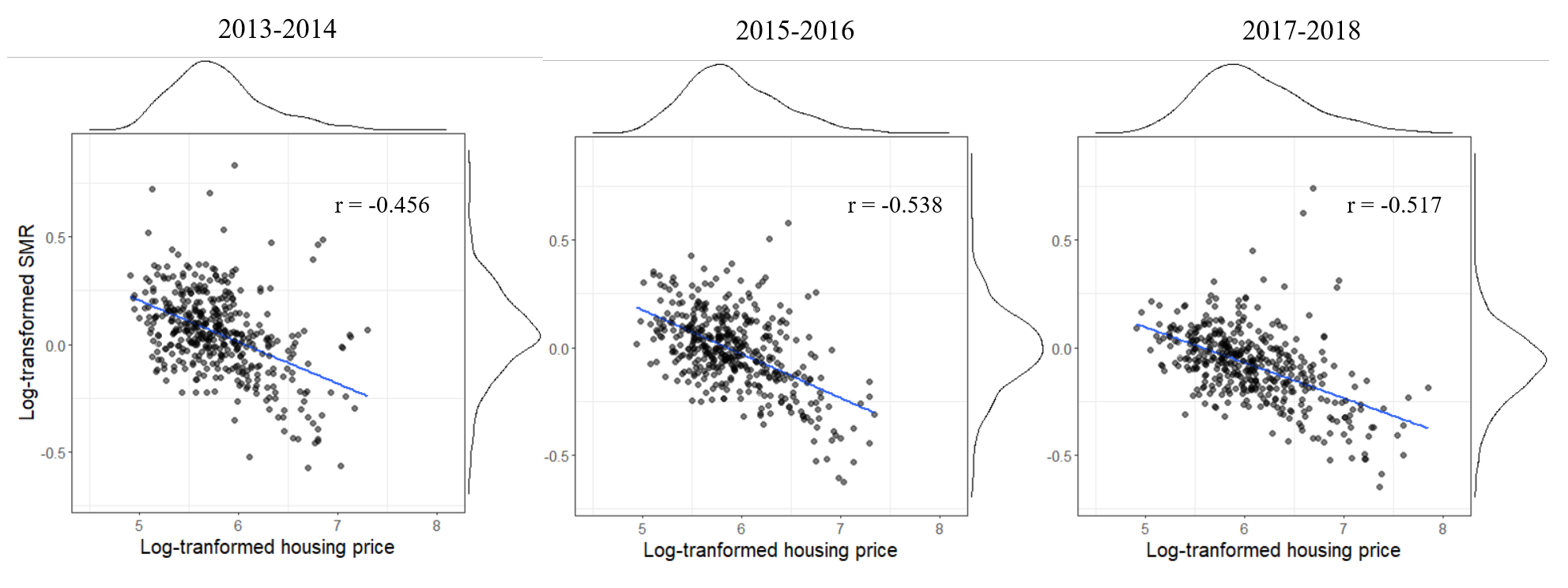


Fig. A.4. The marginal and joint distribution of log-transformed housing prices and SMR, and their Pearson correlation coefficients (r) during the study period.

*Notes.* SMR = Standardized mortality ratio.


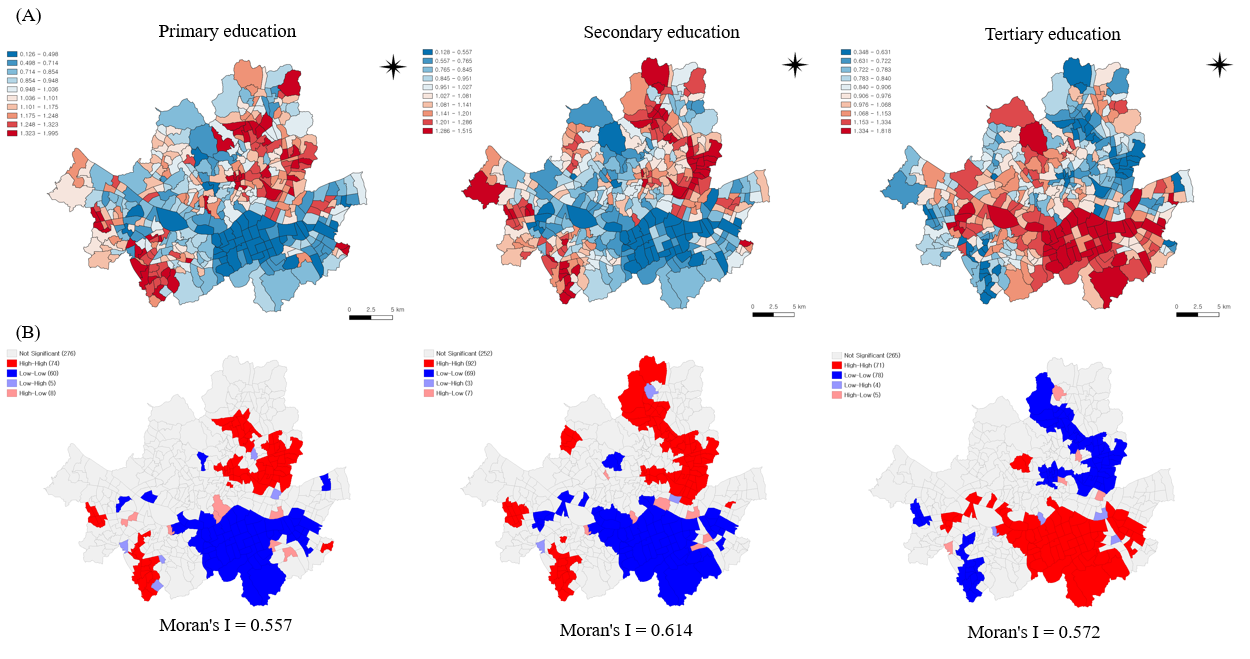


Fig. A.5. Mapped distribution (A) and spatial patterning (B) of educational composition (primary/secondary/tertiary education) by neighborhood in Seoul in 2015.

**Table A.1.** Correlation matrix between educational composition and housing price of neighborhoods in Seoul.

|  | Primary education | Secondary education | Tertiary education | Housing price |
| --- | --- | --- | --- | --- |
| Primary education | 1.000 | 0.910 | -0.805 | -0.690 |
| Secondary education | 0.910 | 1.000 | -0.845 | -0.794 |
| Tertiary education | -0.805 | -0.845 | 1.000 | 0.741 |
| Housing price | -0.690 | -0.794 | 0.741 | 1.000 |


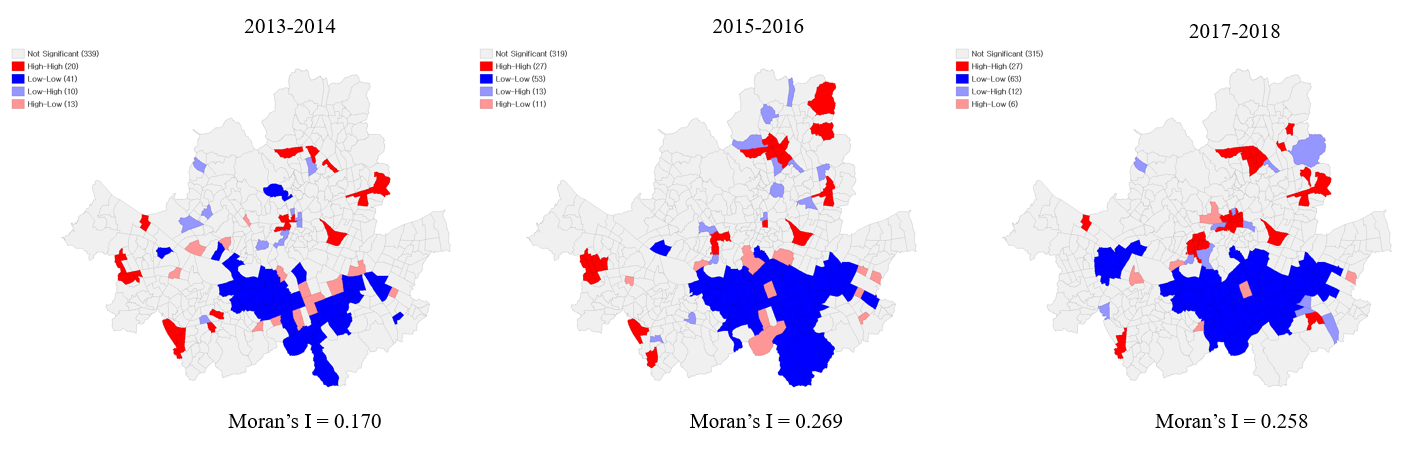


Fig.A.6. Spatial distribution and clustering degree of z-transformed residuals when SMR is calculated using a non-spatial Poisson model.

Table A.2. P-values of simple and robust Lagrange (LM) tests for spatial error and spatial lag model by period

|  | LM error test | LM lag test | Robust LM error test | Robust LM lag test |
| --- | --- | --- | --- | --- |
| 2013–2014 |  |  |  |  |
| Model 3 | 0.695 | 0.510 | 0.044 | 0.036 |
| 2015–2016 |  |  |  |  |
| Model 3 | 0.695 | 0.217 | 0.013 | 0.006 |
| Model 4 | 0.609 | 0.896 | 0.300 | 0.363 |
| 2017–2018 |  |  |  |  |
| Model 3 | 0.037 | 0.002 | 0.553 | 0.021 |

*Notes.* Housing price, poverty rate, population density, business workers density, number of nearby subway stations, number of physicians per 1,000 population, PM10 annual concentration, park area per person, and local tax amount were adjusted in Model 3.

The educational composition of the neighborhood was further controlled in Model 4.


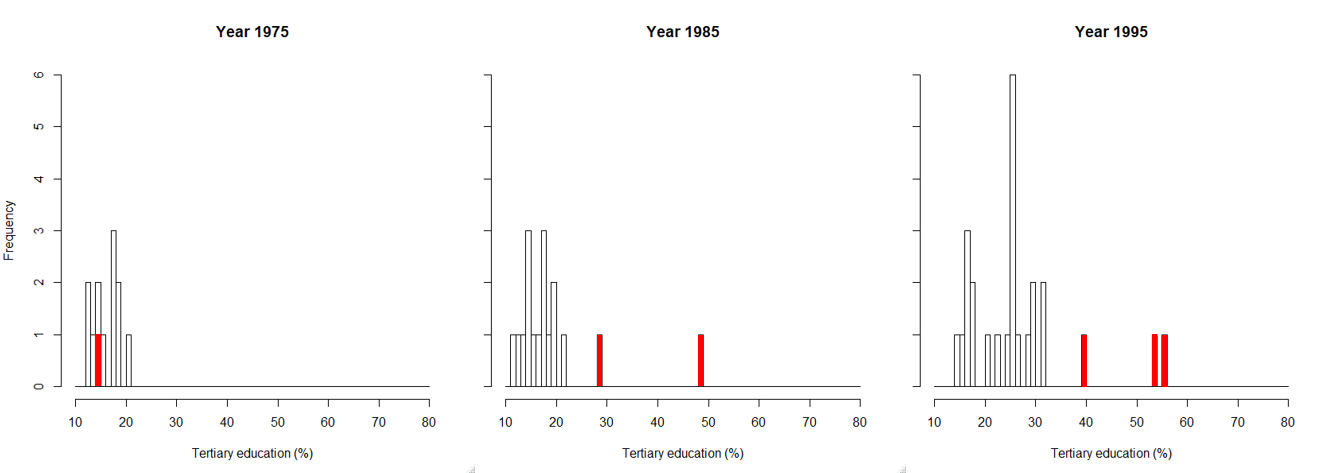


Fig.A.7. Changes in the district-level proportion of tertiary education in Seoul: Findings from census data, 1975–1995

*Notes.* Red bars represent districts belonging to the southeastern part of Seoul (Gangnam area).
